# Barotrauma in COVID-19 acute respiratory distress syndrome: retrospective analysis of the COVADIS prospective multicenter observational database

**DOI:** 10.1186/s12871-023-02093-1

**Published:** 2023-04-27

**Authors:** Nicolas Serck, Michael Piagnerelli, Jean Loup Augy, Filipo Annoni, Gregoire Ottavy, Romain Courcelle, Giuseppe Carbutti, Francois Lejeune, Christophe Vinsonneau, Bertrand Sauneuf, Laurent Lefebvre, Julien Higny, David Grimaldi, Jean-Baptiste Lascarrou

**Affiliations:** 1 Unité de soins intensifs, Clinique Saint Pierre, Ottignies, Belgium; 2 Intensive Care. CHU-Charleroi, Marie Curie, Université Libre de Brussels, 140, chaussée de Bruxelles, Charleroi, 6042 Belgium; 3 Médecine Intensive Réanimation, Hôpital Européen Georges Pompidou, Paris, France; 4 Soins Intensifs, H.UB, Hôpital Erasme, Université Libre de Bruxelles, Brussels, Belgium; 5 Médecine Intensive Réanimation, CHU Nantes, 30 Boulevard Jean Monnet, Nantes Cedex 9, 44093 France; 6 Unité de soins intensifs, Centres Hospitaliers de Jolimont, La Louvière, Belgium; 7 Unité de soins intensifs, CHR Mons-Hainaut, Mons, Belgium; 8 Unité de soins intensifs, Clinique Notre Dame de Grâce, Gosselies, Belgium; 9 Service de Médecine Intensive Réanimation, Unité de Sevrage Ventilatoire et Réhabilitation, Centre Hospitalier de Béthune, 27 Rue Delbecque, Beuvry, 62660 France; 10 Réanimation - Médecine Intensive, Centre Hospitalier Public du Cotentin, Cherbourg-en-Cotentin, BP208, 50102 France; 11 Réanimation polyvalente, Centre Hospitalier du pays d’Aix, Aix en Provence, France; 12 Unité de soins intensifs, CHU Dinant Godinne, site Dinant, Belgium

**Keywords:** COVID-19, Mechanical ventilation, Barotrauma, Pneumothorax, Pneumomediastinum

## Abstract

**Background:**

Despite evidence suggesting a higher risk of barotrauma during COVID-19-related acute respiratory distress syndrome (ARDS) compared to ARDS due to other causes, data are limited about possible associations with patient characteristics, ventilation strategy, and survival.

**Methods:**

This prospective observational multicenter study included consecutive patients with moderate-to-severe COVID-19 ARDS requiring invasive mechanical ventilation and managed at any of 12 centers in France and Belgium between March and December 2020. The primary objective was to determine whether barotrauma was associated with ICU mortality (censored on day 90), and the secondary objective was to identify factors associated with barotrauma.

**Results:**

Of 586 patients, 48 (8.2%) experienced barotrauma, including 35 with pneumothorax, 23 with pneumomediastinum, 1 with pneumoperitoneum, and 6 with subcutaneous emphysema. Median time from mechanical ventilation initiation to barotrauma detection was 3 [0–17] days. All patients received protective ventilation and nearly half (23/48) were in volume-controlled mode. Barotrauma was associated with higher hospital mortality (*P* < 0.001) even after adjustment on age, sex, comorbidities, PaO_2_/FiO_2_ at intubation, plateau pressure at intubation, and center (*P* < 0.05). The group with barotrauma had a lower mean body mass index (28.6 ± 5.8 vs. 30.3 ± 5.9, *P* = 0.03) and a higher proportion of patients given corticosteroids (87.5% vs. 63.4%, *P* = 0.001).

**Conclusion:**

Barotrauma during mechanical ventilation for COVID-19 ARDS was associated with higher hospital mortality.

## Introduction

The severe acute respiratory syndrome coronavirus 2 (SARS-CoV-2) pandemic has infected at least 460 million people worldwide, and the official count of 6 million deaths is probably an underestimation [1]. The most common cause of death in coronavirus virus disease 2019 (COVID-19) is acute respiratory distress syndrome (ARDS) with hypoxemic respiratory failure [2]. Among patients admitted for COVID-19, 8–32% require admission to the intensive care unit (ICU) [3, 4] and 19% are placed on invasive mechanical ventilation [4].

Barotrauma from mechanical ventilation is defined clinically as alveolar rupture manifesting as pneumomediastinum, pneumothorax, pneumopericardium, and/or subcutaneous emphysema [5]. The pressures and volumes applied by the ventilator play a key role, although factors that weaken the alveolar wall may also be involved [5]. Barotrauma is a well-documented complication of non-COVID-19 viral ARDS requiring mechanical ventilation for whatever reason [5]. Protective ventilation strategies that limit ventilation volumes and pressures are recommended to avoid these complications, notably in patients with ARDS [6]. Compared to other forms of ARDS, COVID-19 ARDS has been described as atypical given the higher lung compliance and gas volume at a given PaO_2/_/FiO_2_ ratio [7]. Another atypical feature may be a higher risk of barotrauma: a literature review published in March 2022 showed barotrauma in 14.7% of COVID-19 patients compared to 6.3% of patients with ARDS due to other causes [8]. Other studies found barotrauma in up to 26.7% of patients [9, 10]. Also, rare cases of barotrauma have been reported in spontaneously breathing patients with COVID-19 [11, 12].

The primary objective of this retrospective analysis of the prospective multicenter observational COVADIS study was to determine whether barotrauma was associated with hospital mortality. The secondary objectives were to evaluate the incidence, risk factors, and other outcomes of barotrauma.

## Patients and methods

This report complies with STROBE guidelines [13].

### Study design and patients

This was a retrospective analysis of the data from the COVADIS observational cohort study. COVADIS prospectively included patients admitted between March and December 2020 to any of 12 ICUs, including 7 in Belgium and 5 in France [14–18]. Inclusion criteria were age older than 18 years, moderate-to-severe ARDS according to the Berlin definition [19] (PaO_2_/FiO_2_ < 200 mmHg with positive end-expiratory pressure ≥ 5 mmHg during invasive mechanical ventilation), and positive COVID-19 reverse-transcriptase polymerase-chain-reaction test on a sample from any site. Patients with negative COVID-19 polymerase chain reaction tests were not included even when they had computed tomography abnormalities typical for COVID-19. Non-inclusion criteria were cardiac arrest before ICU admission, extracorporeal membrane oxygenation within 24 h after ICU admission, Gold stage III or IV chronic obstructive pulmonary disease, and home oxygen therapy.

### Data collection

Between March 10 and December 31, 2020, consecutive COVID-19 patients admitted to the participating ICUs were screened for eligibility, and those who met the inclusion and non-inclusion criteria were enrolled in the cohort. The investigator in each ICU used an electronic case-report form (Castor EDC, Amsterdam, The Netherlands) to record the following for each patient: demographics; medical history; Charlson Comorbidity Index [20] with addition of chronic hypertension; and the Sequential Organ Failure Assessment score at ICU admission [21]. Recorded data describing the ICU management included mechanical ventilation settings and duration; use of advanced treatments for acute respiratory failure (neuromuscular blocking agents, inhaled pulmonary vasodilators, prone positioning, and extracorporeal membrane oxygenation); use of antivirals, interleukin-6-receptor antagonists, and corticosteroids, with time from symptom onset to initiation; acute kidney injury; acute cardiac injury defined as troponin elevation above 10 times the upper limit of normal; use of norepinephrine and/or epinephrine and/or vasopressin; and occurrence of pulmonary embolism and/or deep vein thrombosis. Cases of barotrauma with their characteristics were collected. Barotrauma was defined as the presence of air outside the pleural aspect of the lung and included pneumothorax, pneumomediastinum, pneumopericardium, pneumoperitoneum, and subcutaneous emphysema. Patients were not screened routinely for barotrauma during the study period. The strategy for diagnosing barotrauma was at the discretion of each managing physician and could include physical examination, transthoracic and/or transesophageal echocardiography, chest radiography, chest computed tomography, and/or abdominal computed tomography. We defined baseline (T0) as the day of ICU admission.

### Outcomes

The primary objective was to assess whether barotrauma was associated with ICU mortality, censored on day 90, which was therefore the primary outcome measure. The secondary objectives were to determine the incidence, risk factors, and other outcomes associated with barotrauma.

### Statistical analysis

Based on two studies of ARDS, we planned to include at least 500 patients to obtain at least 30 patients with barotrauma [22, 23].

Continuous variables were described as mean ± SD or median [IQR] and compared by applying Student’s *t* test if normally distributed and the Wilcoxon rank-sum test otherwise. Categorical variables were described as n (%) and compared using the chi-square test or Fisher’s exact test, as appropriate.

A pre-planned adjusted mixed multivariable analysis was performed using a generalized mixed model to identify associations linking ICU mortality (primary outcome, censored on day 90) to barotrauma. Adjustment variables were age, sex, baseline plateau pressure, baseline PaO_2_/FiO_2_, Charlson Comorbidity Index, and center [15]. The Hosmer-Lemeshow test and visual inspection of residuals were chosen to check the quality of the model.

No imputation was performed for missing data. *P* values < 0.05 were considered significant.

All analyses were performed using Stata software version 16 (StataCorp, College Station, TX).

## Results

### Baseline characteristics

Of the 586 included patients, 48 (8.1%) experienced barotrauma. Table 1 reports their main features at baseline.

**Table 1:** Main patient characteristics and treatments

Characteristics	Overall N = 586	Barotrauma N = 48	No barotrauma N = 538	*P* value
Age, y, mean ± SD	64 ± 11	63 ± 10	64 ± 11	0.49
Males, n (%)	439 (74.9)	38 (79.1)	401 (74.5)	0.49
BMI, kg/m², mean ± SD	30.2 ± 5.9	28.6 ± 5.8	30.3 ± 5.9	0.03
Chronic hypertension, n (%)	352 (60.0)	27 (56.2)	325 (60.4)	0.56
CCI, median [IQR]	1 [0–3]	1 [0–3]	1 [0–3]	0.87
Symptom onset to ICU admission, days, median [IQR]	8 [6–10]	7 [5–12]	8 [6–10]	0.68
Symptom onset to eMV initiation, days, median [IQR]	9 [7–12]	9 [7–16]	9 [7–12]	0.17
Antiviral treatment, n (%)				
- Lopinavir/ritonavir - Hydroxychloroquine - Macrolides - Remdesivir	43 (7.3) 153 (26.1) 173 (29.5) 27 (4.6)	1 (2.1) 1 (2.1) 8 (16.7) 0	42 (7.8) 152 (28.3) 165 (30.7) 27 (5.0)	0.14 < 0.001 0.04 0.11
Corticosteroids, n (%)	383 (65.3)	42 (87.5)	341 (63.4)	0.001
Corticosteroids within 48 h after ICU admission, n (%)	222 (37.8)	26 (54.1)	196 (36.4)	0.58
Symptom onset to corticosteroid initiation, days, median [IQR] (N = 383)	7 [5–10]	7 [5–9]	7 [5–10]	0.09
Tocilizumab, n (%)	23 (3.9)	3	20	0.39
SOFA score^a^, median [IQR]	6 [3–8]	6 [3–7]	6 [3–8]	0.41
V_T_, mL/kg IBW, median [IQR]	6.3 [5.8–7.1]	6.3 [5.8–7.3]	6.3 [5.8–7.1]	0.62
Total PEEP (cmH_2_O), median [IQR]	10 [8–12]	10 [6–13]	10 |8–12]	0.42
Plateau pressure (cmH_2_O), median [IQR]	24 [20–26]	24 [20–28]	24 [20–26]	0.38
PaO_2_/FiO_2_, median [IQR]	106 [77–143]	100 [75–130]	106 [78–144]	0.20
Static compliance, mL/cmH_2_O, median [IQR]	34 [28–44]	34 [24–46]	34 [28–44]	0.51
Neuromuscular blockade, n (%)	523 (89.2)	44	479	0.59
Inhaled nitric oxide, n (%)	22 (3.7)	0	22	0.15
Prone position, n (%)	489 (83.4)	44	445	0.11
VV-ECMO, n (%)	65 (11.1)	8	57	0.20

^a^determined at admission to the intensive care unit

BMI: body mass index; CCI: Charlson’s Comorbidity Index; eMV: endotracheal mechanical ventilation; ICU: intensive care unit; SOFA: Sequential Organ Failure Assessment; V_T_: tidal volume; IBW: ideal body weight, PEEP: positive end-expiratory pressure; PaO_2_/FiO_2_: ratio of arterial partial pressure of oxygen over fraction of inspired oxygen; VV-ECMO: veno-venous extracorporeal membrane oxygenation

### Barotrauma and association with day-90 mortality

Table 1 compares the baseline features in patients with vs. without barotrauma. Barotrauma manifested as pneumothorax (n = 35, 6%), pneumomediastinum (n = 23, 4%), and/or pneumoperitoneum (n = 1, < 1%); no patient had pneumopericardium. Subcutaneous emphysema developed in 6 (1%) patients, all of whom had at least one of the above-listed manifestations. Median time from invasive mechanical ventilation initiation to barotrauma was 3 [0–17] days. Table 2 reports the ventilator settings at barotrauma detection. PaO_2_/FiO_2_ within 12 h before barotrauma detection was 136 [90–180]. Of the 35 patients with pneumothorax, 24 (50%) required pleural drainage and 1 (2%) surgery. Of the 48 patients with barotrauma, only 3 (6%) required no intervention and 43 required one or more interventions among the following: pleural drainage (n = 24, 50%), ventilation mode change (n = 14, 29%), sedation regimen change (n = 10, 21%), surgery (n = 1, 2%), and other interventions (e.g., neuromuscular blockade or cardiac-arrest resuscitation) (n = 5, 10%). Table 3 compares the other outcomes and survival in patients with vs. without barotrauma.

**Table 2:** Ventilator settings at barotrauma detection in the 48 patients with barotrauma

Ventilator settings, n (%)	n patients (%)
During endotracheal mechanical ventilation	
Volume-controlled	23(47.9)
Pressure-assisted	7(14.6)
Pressure-controlled	3(6.2)
Airway pressure release ventilation	5(10.4)
Weaned off endotracheal mechanical ventilation	
One-level positive pressure	3(6.2)
High-flow nasal cannula	4(8.3)
Standard oxygen	3(6.2)

**Table 3:** Outcomes in patients with and without barotrauma

	Barotrauma N = 48	No barotrauma N = 536	*P* value
**Renal replacement therapy, n (%)**	33 (68.7)	316 (58.9)	0.18
**Creatinine peak, µmol/l, median [IQR]**	164 [114–340]	131 [79–319]	
**Acute cardiac injury, n (%)**	8 (16.6)	6 (1.1)	0.14
**Vasopressors during ICU stay, n (%)**	43 (89.6)	433 (80.7)	0.13
**Pulmonary embolism, n (%)**	12 (25.0)	58 (10.8)	0.004
**Deep vein thrombosis, n (%)**	3 (6.2)	34 (6.3)	0.98
**ICU discharge alive, n (%)**	16 (33.3)	291 (54.2)	0.005
**eMV duration, days, median [IQR]**	19 [11–33]	14 [8–24]	0.01
**ICU stay length, days, median [IQR]**	20 [12–36]	18 [10–29]	0.09
**Hospital discharge alive, n (%)**	13 (27.0)	289 (53.9)	< 0.001
**Hospital stay length, days, median [IQR]**	22 [13–43]	22 [12–38]	0.54

ICU: intensive care unit; eMV: endotracheal mechanical ventilation

**Fig. 1:**
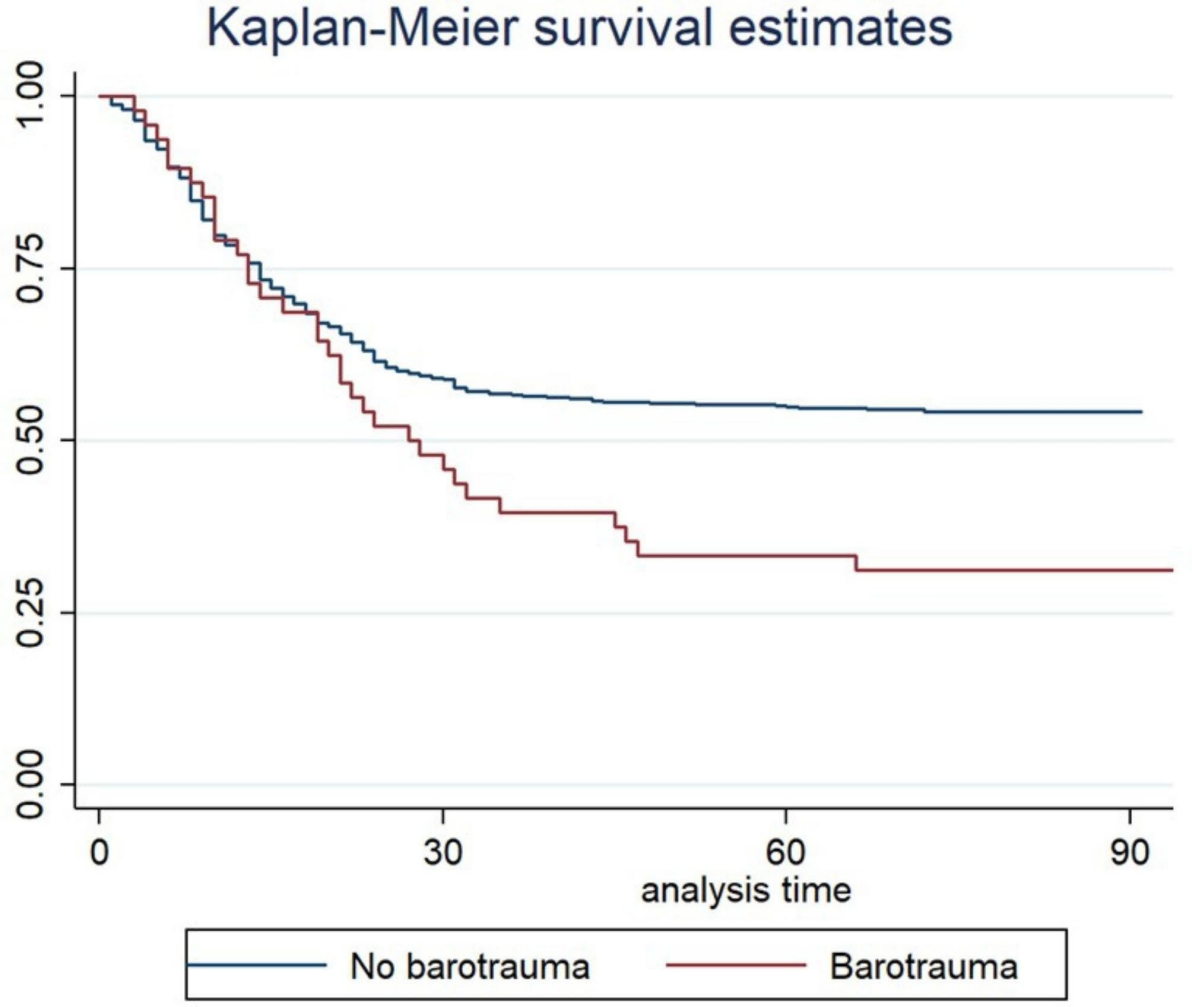
Kaplan-Meier survival plots in the groups with and without barotrauma

**Fig. 2:**
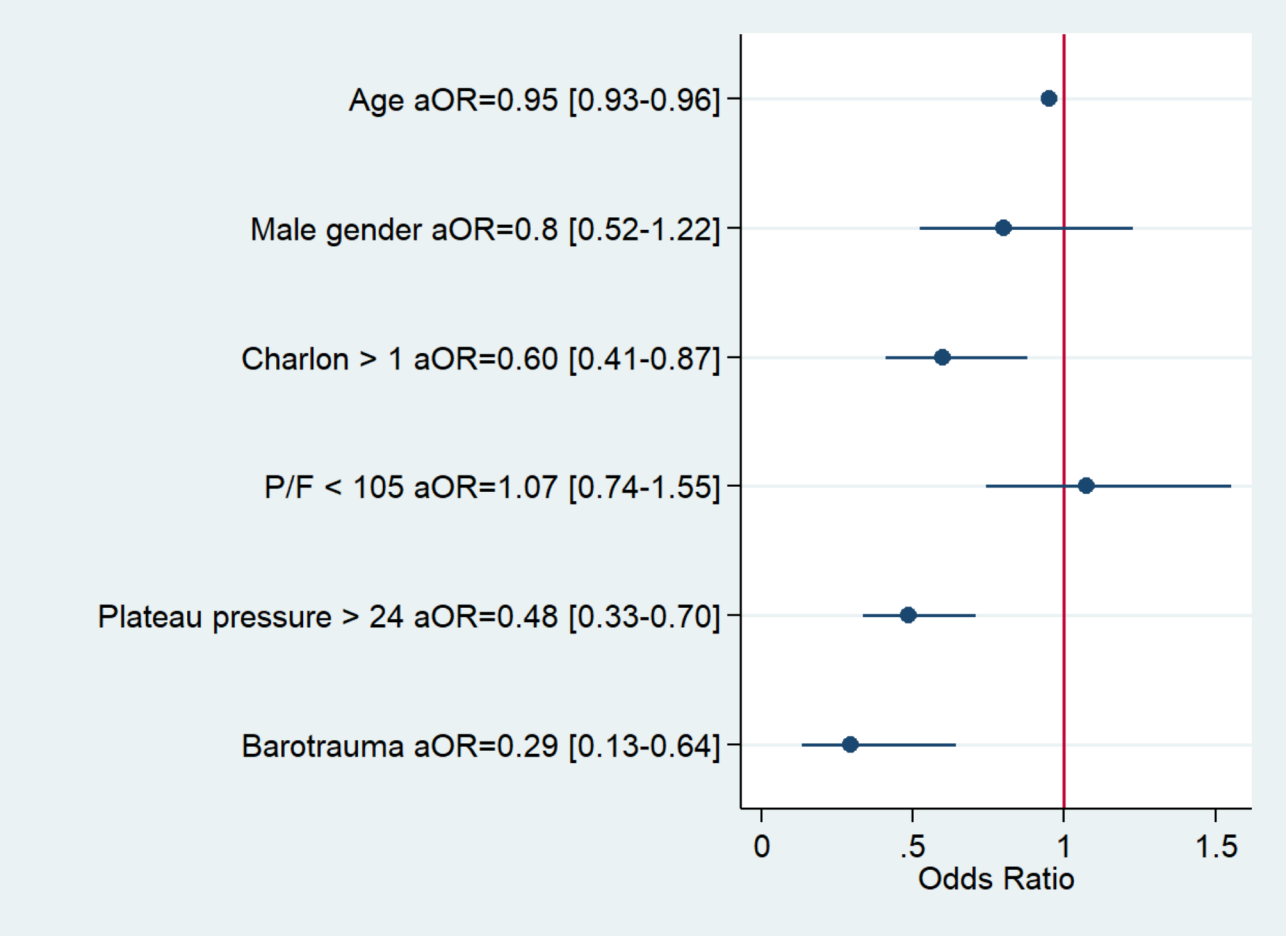
Forest plot of factors analyzed for association with day-90 mortality

Tables 1 and 3 compare the baseline features and outcomes, respectively, in patients with vs. without barotrauma. Figure 1 is the Kaplan-Meier plot of survival censored on day 90 in each group. After adjustment on age, male sex, Charlson Comorbidity Index, PaO_2_/FiO_2_ at intubation, plateau pressure at intubation, and center, barotrauma was significantly and independently associated with higher day-90 mortality (Fig. 2).

## Discussion

Of 586 patients who required mechanical ventilation for moderate-to-severe COVID-19 ARDS, 48 (8.2%) experienced barotrauma. Only 6% of patients with barotrauma required no additional interventions to treat this event, and half required pleural drainage. In the multivariable analysis adjusted for potential confounders, barotrauma was independently associated with death before hospital discharge.

The 8.2% frequency of barotrauma in our patients is within the reported range of 3.5–8.6% for all-cause ARDS [23–26] and is lower than the 26.7% frequency reported in COVID-19 ARDS very early in the pandemic (March and April 2020) [27]. The lower frequency in our population may be ascribable to the uniformity of the patient population with moderate-to-severe ARDS, with high adherence to neuromuscular blockade infusion [16] and prone positioning [15]. Protective ventilation, when properly applied, decreases the risk of barotrauma. Nonetheless, even with protective ventilation, barotrauma in COVID-19 ARDS has occurred in 17% [28], 24% [27], and 40% [29] of patients. In a study comparing non-COVID-19 to COVID-19 ARDS managed with protective ventilation, the incidences of barotrauma were 1.9% and 13.6%, respectively [30]. These high frequencies suggest the involvement of factors other than the ventilation pattern in the development of barotrauma during COVID-19 ARDS, particularly given the higher lung compliance in COVID-19 ARDS compared to other causes of ARDS [7].

Direct damage to the alveolar wall induced by SARS-CoV-2 deserves consideration as a possible contributor to barotrauma. Consistent with this possibility are several reports of air leakage outside the alveoli in patients with COVID-19 pneumonia who were not receiving ventilatory assistance [12]. If this extra-alveolar air is not related to high inspiratory pressures or to hyperinflation linked to excessive tidal volumes, another cause must be sought. Macklin first studied the causes of extra-alveolar air, in the 1940s [31]. The Macklin effect has been defined as a linear collection of air contiguous to the bronchovascular sheaths on lung parenchyma-windowed computed tomography images [32]. Macklin stated that air released by alveolar destruction migrated via dissection of the bronchovascular tree from the alveoli to the pulmonary hilum. Alveolar destruction can be caused by barotrauma (high inspiratory pressures or hyperinflation) or by direct damage to the alveoli. However, lung-protection ventilation parameters designed to prevent extra-alveolar air were used in our patients. This leaves direct alveolar damage by the virus as the likely cause of alveolar destruction [33]. Interestingly, the Macklin effect was recently identified by baseline computed tomography in 33 of 37 COVID-19 patients who subsequently experienced pneumothorax and/or pneumomediastinum, the median time interval being 8.5 [1–18] days [34].

Second, differences have been reported between ARDS due to COVID-19 vs. other causes, including higher lung compliance and lung gas volumes [35]. Differences may also exist in damage to the alveolar wall, notably given the very high degree of inflammation, with a cytokine storm, in COVID-19 [36].

Third, corticosteroids increase tissue fragility [37] and may therefore weaken the alveolar wall. In a comparison of the first and second COVID-19 waves in Italy, 14 of 2635 non-intubated patients experienced pneumothorax or pneumomediastinum, including 1 during the first and 13 during the second wave. The main treatment difference was the widespread use of corticosteroids during the second wave [38]. Thus, all 13 patients identified during the second wave were on corticosteroid therapy, whereas the single patient during the first wave was not. In interstitial lung disease, an association linking corticosteroid therapy to pneumothorax has been reported [39]. In our study, the proportion of patients given corticosteroid therapy was significantly higher in the group with vs. without barotrauma. The times from symptom onset and from intubation to corticosteroid initiation were not significantly different between the two groups.

Fourth, COVID-19 is a thrombogenic disease, and thromboprophylaxis is now a key component of its management [40]. Pulmonary embolism in our cohort was significantly more common among patients with vs. without barotrauma. Conceivably, microvascular dysfunction might contribute to barotrauma in COVID-19 [41]. A word of caution is in order, however: whether these specific characteristics of COVID-19 compared to other causes of ARDS deserve a change in ventilation strategies is unclear, as similar respiratory mechanics have been reported [42].

Interestingly, of the 35 patients with pneumothorax, 24 (68%) required pleural drainage, a proportion similar to that noted in a multicenter case-control study (73/110, 66%) [43]. Apart from pneumothorax drainage, barotrauma had several consequences on patient management. In our study, the ventilation pattern was changed in over a quarter of patients and the sedation regimen in over a fifth of patients. Changes in ventilator settings after barotrauma aim to further protect the alveoli. The resulting decreased ability to perform aggressive recruitment maneuvers may increase invasive mechanical ventilation duration and decrease survival [26]. Increased sedation is designed to minimize asynchronies potentially associated with barotrauma but is associated with longer invasive mechanical ventilation times. The changes in ventilator settings and sedation probably explain the significantly longer invasive mechanical ventilation duration in our barotrauma group.

The limitations of our study include the availability of ventilation parameters only for the time of intubation and the time of barotrauma detection. Consequently, we were unable to evaluate potential links between the overall protective ventilation strategy and barotrauma. Second, the design was observational, with treatment decisions at the discretion of the managing physicians. Finally, the patients were included during the first ten months of the pandemic, i.e., the first and second waves in France. Whether the frequency and risk factors of barotrauma have changed with the emergence of new SARS-CoV-2 variants and with the major changes in COVID-19 management during this period cannot be determined from our data.

## Conclusion

In patients with moderate-to-severe COVID-19 ARDS requiring invasive mechanical ventilation, barotrauma was significantly associated with higher hospital mortality. Barotrauma was associated with longer invasive mechanical ventilation duration, pulmonary embolism, and corticosteroid therapy. The mechanism of barotrauma occurring in COVID-19 despite protective ventilation and, more specifically, the possible role for corticosteroid therapy deserve investigation.

## Data Availability

The study database will be made available upon request to the corresponding author after approval of the study request protocol by our institutional review board.

## Abbreviations

ARDS: acute respiratory distress syndrome
COVID-19: coronavirus disease 2019
ICU: intensive care unit
PaO_2_/FiO_2_: ratio of arterial partial oxygen pressure over fraction of inspired oxygen
SARS-CoV-2: severe acute respiratory syndrome coronavirus 2
