# Correction: Barotrauma in COVID-19 acute respiratory distress syndrome: retrospective analysis of the COVADIS prospective multicenter observational database

**DOI:** 10.1186/s12871-023-02175-0

**Published:** 2023-06-20

**Authors:** Nicolas Serck, Michael Piagnerelli, Jean Loup Augy, Filippo Annoni, Gregoire Ottavy, Romain Courcelle, Giuseppe Carbutti, Francois Lejeune, Christophe Vinsonneau, Bertrand Sauneuf, Laurent Lefebvre, Julien Higny, David Grimaldi, Jean-Baptiste Lascarrou

**Affiliations:** 1 Unité de soins intensifs, Clinique Saint Pierre, Ottignies, Belgium; 2 Intensive Care. CHU-Charleroi, Marie Curie, Université Libre de Brussels, 140, Chaussée de Bruxelles, Charleroi, 6042 Belgium; 3 Médecine Intensive Réanimation, Hôpital Européen Georges Pompidou, Paris, France; 4 Soins Intensifs, H.UB, Hôpital Erasme, Université Libre de Bruxelles, Brussels, Belgium; 5 Médecine Intensive Réanimation, CHU Nantes, 30 Boulevard Jean Monnet, Nantes Cedex 9, 44093 France; 6 Unité de soins intensifs, Centres Hospitaliers de Jolimont, La Louvière, Belgium; 7 Unité de soins intensifs, CHR Mons-Hainaut, Mons, Belgium; 8 Unité de soins intensifs, Clinique Notre Dame de Grâce, Gosselies, Belgium; 9 Service de Médecine Intensive Réanimation, Unité de Sevrage Ventilatoire Et Réhabilitation, Centre Hospitalier de Béthune, 27 Rue Delbecque, Beuvry, 62660 France; 10 Réanimation - Médecine Intensive, Centre Hospitalier Public du Cotentin, BP208, Cherbourg-en-Cotentin, 50102 France; 11 Réanimation Polyvalente, Centre Hospitalier du Pays d’Aix, Aix en Provence, France; 12 Unité de soins intensifs, CHU Dinant Godinne, Site Dinant, Belgium

**Correction: BMC Anesthesiol 23, 138 (2023)**

**https://doi.org/10.1186/s12871-023-02093-1**

Following publication of the original article [1], the authors identified an error in the author name of Filippo Annoni.

The incorrect author name is: Filipo Annoni

The correct author name is: Filippo Annoni

The author group has been updated above and the original article [1] has been corrected.
